# S100A11 activates the pentose phosphate pathway to induce malignant biological behaviour of pancreatic ductal adenocarcinoma

**DOI:** 10.1038/s41419-022-05004-3

**Published:** 2022-06-25

**Authors:** Xue Zeng, Hong Guo, Zhuang Liu, Zilan Qin, Yuyang Cong, Naihan Ren, Yuxiang Zhang, Na Zhang

**Affiliations:** Department of Radiation Oncology, Cancer Hospital of China Medical University, Liaoning Cancer Hospital & Institute, Cancer Hospital of Dalian University of Technology, Shenyang, 110042 PR China

**Keywords:** Oncogenes, Cancer metabolism

## Abstract

Pancreatic ductal adenocarcinoma (PDAC) is one of the most refractory malignancies and has a poor prognosis. In recent years, increasing evidence has shown that an imbalance of metabolism may contribute to unrestricted pancreatic tumour progression and that the pentose phosphate pathway (PPP) plays a pivotal role in cellular metabolism. S100A11 has been shown to regulate multiple biological functions related to the progression and metastasis of various cancer types. However, the exact mechanisms and prognostic value of S100A11 in PDAC remain unclear. Here, we found that S100A11 expression was increased in PDAC and significantly associated with worse prognosis and disease progression. Mechanistically, S100A11 knockdown suppressed the PPP by impairing nascent mRNA synthesis of TKT (transketolase). The current study also demonstrated that H3K4me3 at the −268/+77 region of the *TKT* promoter was required for its transcriptional activation and S100A11 promoted H3K4me3 loading to the *TKT* promoter by interacting with SMYD3 protein. Taking these findings together, this study provided new insights into the potential value of S100A11 for treating pancreatic cancer, suggesting that it could be a therapeutic target for PDAC patients.

## Introduction

Pancreatic ductal adenocarcinoma (PDAC) is an intractable malignancy with rapid tumour proliferation and early distant metastasis. Dramatic progress has been made in treating PDAC over the last decade. Unfortunately, the effectiveness of existing medical approaches is still limited [1]. Many recent reports have indicated that intracellular metabolism is crucial for limiting the benefit of treatment with anti-cancer therapy [2, 3]. Therefore, there is an urgent need to obtain a better understanding of the causative factors that drive progression and metastasis to improve the survival status of PDAC.

Cancer cells exhibit extraordinary growth advantages under conditions of hypoxia and poor nutrition [4]. The main cause is the change of intracellular energy metabolism. The pentose phosphate pathway (PPP), as a committed step of glucose metabolism, which is required for maintaining the cellular redox state. Cancer cells modulate the flux of the PPP depending on the particular metabolic demand [5, 6]. Activated PPP generates high levels of NADPH and ribonucleotides in cancer cells, which promotes pancreatic tumorigenesis and metastasis [7–9], emphasising the vital role of PPP in pancreatic cancer development.

S100A11 is a member of the S100 family that interacts with calcium and is involved in multiple biological processes, such as cell proliferation, migration and signal transduction [10–12]. Accumulating evidence has shown that high expression of S100A11 may serve as a predictor of an unfavourable prognosis in PDAC [13, 14]. Moreover, as a protein that can enter the nucleus, S100A11 can affect the expression of different genes and thus exert various functions [15, 16]. However, the specific molecular mechanism behind the function of S100A11 in pancreatic cancer remains unclear.

In the present study, we showed that S100A11 expression was increased and positively correlated with disease progression in PDAC. S100A11 downregulation suppressed the malignant biological properties of PDAC cells. In terms of the mechanism involved, H3K4me3 protein was specifically recruited to the promoter of TKT at the region of −268/+77 facilitated by S100A11 interacting with SMYD3, which activated the PPP and further promoted PDAC progression. Therefore, targeting S100A11 should be an attractive option for novel treatments of pancreatic cancer.

## Results

### Overexpression of S100A11 conferred poor survival in patients with PDAC

**Fig. 1:**
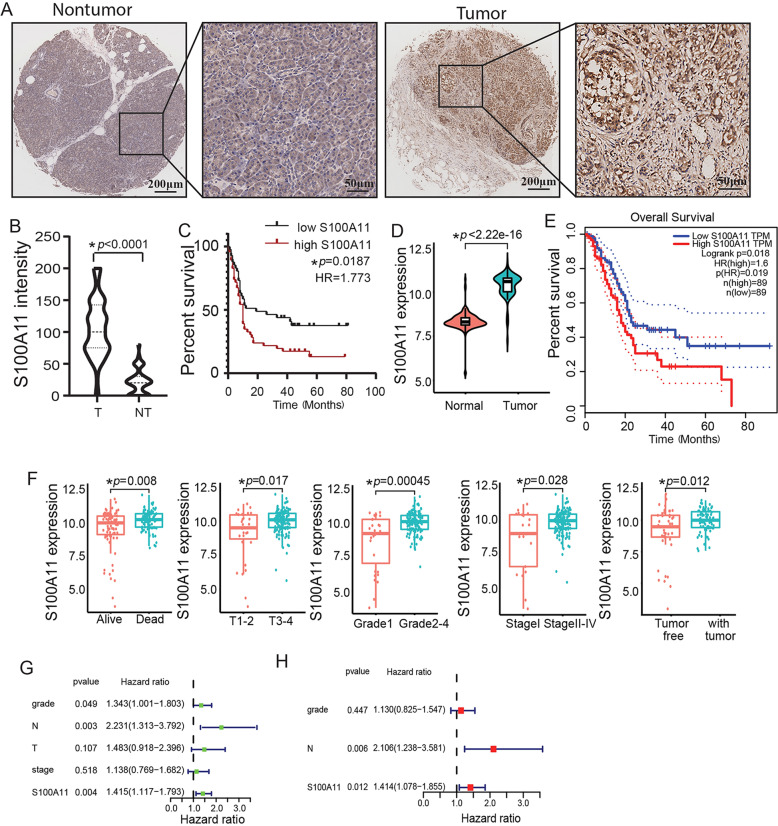
S100A11 expression is upregulated and predicts poor prognosis of patients with PDAC. **A** Analysis of S100A11 protein expression in PDAC and paracancerous tissues through IHC-based TMA (inset: 20× magnified image). **B, D** Violin plots demonstrating the difference in S100A11 expression between PDAC and paired paracancerous samples in the TMA cohort and TCGA database, respectively. **C, E** Kaplan–Meier analyses of the association between S100A11 expression and OS of PDAC from the TMA cohort and TCGA database, respectively. **F** Correlation analysis between the S100A11 expression and clinicopathological characteristics using online TCGA case cohort. **G, H** Univariate and multivariate Cox regression analyses of the relationship between the expression of S100A11 and clinicopathological factors with TCGA database. T, pancreatic cancer tissues. NT, normal paracancerous tissues. *p < 0.05.

In our study, 90 tumour tissues of PDAC and 60 paired paracancerous tissues were used to assess S100A11 protein expression using TMA. Notably, S100A11 was mostly expressed in the nucleus as well as the cytoplasm of pancreatic cancer tissues with strong staining, whereas it was confined to the cytoplasm in normal paracancerous samples with weak intensity (Fig. 1A). Furthermore, S100A11 was significantly upregulated in pancreatic cancer tissues compared with the level in normal paracancerous tissues (Fig. 1B). Kaplan-Meier analysis showed that the OS of patients was inversely associated with the level of S100A11 expression (Fig. 1C). Similar results were obtained by TCGA analysis, with the expression level of S100A11 in tumour tissues being significantly higher than in paracancerous tissues (Fig. 1D) and there being poor prognosis in the cohort with high S100A11 expression (Fig. 1E). Correlation analyses clarified the correlations between S100A11 expression and clinicopathological factors. Those who died of pancreatic cancer, and had higher T stage (T3–4), pathological grade (G3–4) and clinical stage (II–IV), or had a tumour in the pancreas body among TCGA cohort had significantly increased risk scores (Fig. 1F). By univariate Cox analysis, pathological grade, lymph node stage and S100A11 expression were risk factors associated with poor OS (Fig. 1G). High S100A11 expression and lymph node stage were parameters independently associated with prognosis when in the multivariate Cox analysis (Fig. 1H). The above findings further demonstrate that S100A11 might act as an unfavourable prognostic factor in PDAC.

### S100A11 promoted malignant properties of PDAC cells

**Fig. 2:**
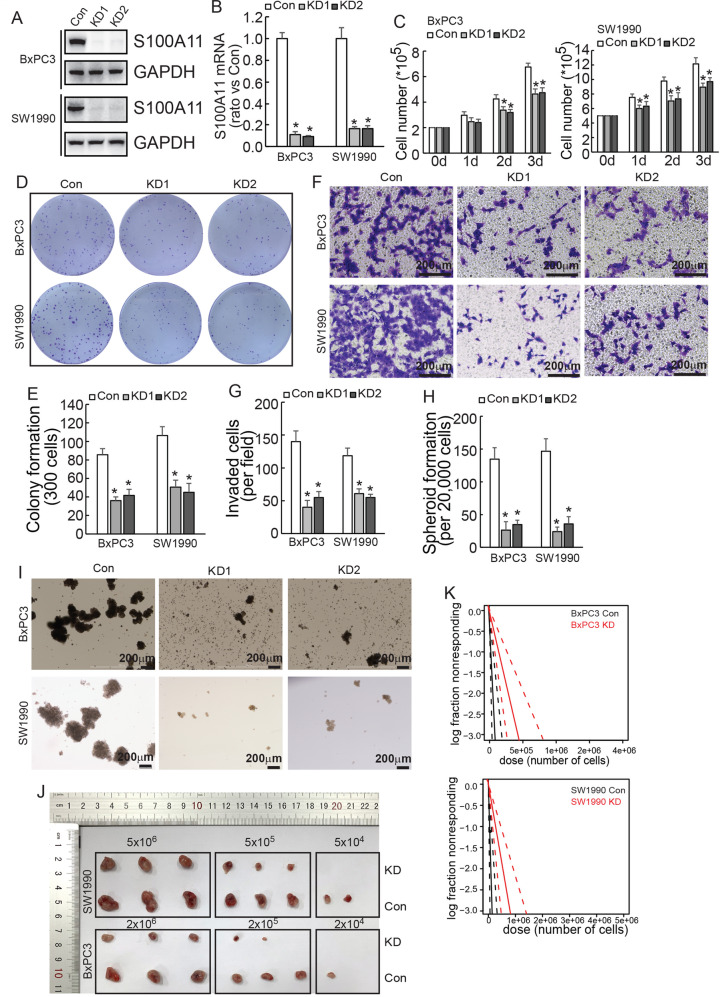
S100A11 facilitates cell proliferation, migration and stem cell potential of PDAC cells. **A, B** S100A11 was knocked down using the CRISPR-Cas9 system. The knockdown efficiency was confirmed by western blotting (**A**) and quantative real-time PCR (**B**) in PDAC cells. **C** Control or S100A11-knockdown PDAC cells were plated on six-well plates and cell numbers were counted daily for 3 days. **D, E** S100A11 knockdown inhibited the proliferation of PDAC cells, as detected by colony formation assay. **F, G** Transwell assay indicated that S100A11 knockdown decreased the invasiveness of PDAC cells. **H, I** The spheroid formation assay revealed that knockdown of S100A11 impaired the ability of PDAC cells to form spheroids. **J** Representative tumours originating from control or S100A11-knockdown PDAC cells are shown. **K** CSC frequency in PDAC from an in vivo limiting dilution assay was calculated using ELDA software. *p < 0.05. Error bars indicate mean ± SD. Con, PDAC cells transfected with S100A11 control sequence; KD1, PDAC cells transfected with S100A11-specific knockdown sequence 1; KD2, PDAC cells transfected with S100A11-specific knockdown sequence 2.

To investigate the regulatory role of S100A11 in proliferation, invasion and spheroid formation in pancreatic cancer, S100A11 was knocked down using CRISPR/Cas9 system in BxPC3 and SW1990 cells (Fig. 2A, B). Cell number count (Fig. 2C) and colony formation assay (Fig. 2D, E) demonstrated that knockdown of S100A11 inhibited the proliferation of PDAC cells. Moreover, cell invasiveness was also significantly decreased (Fig. 2F, G). Because several studies have reported that S100A11 was associated with tumour stem cells, we tested its effect on spheroid formation and observed that its knockdown markedly impaired the ability to form spheroids (Fig. 2H, I).

ELDA clarified the effect of S100A11 on cancer stem cell (CSC) frequency in vivo. When higher numbers of PDAC cells were inoculated (5 × 10^6^ SW1990 and 2 × 10^6^ BxPC3), the number of formed tumours did not differ significantly, but S100A11-knockdown cells had significantly smaller volume than upon injection with control cells (*n* = 3). Remarkably, upon the inoculation of a lower number of cells (5 × 10^4^ SW1990 and 2 × 10^4^ BxPC3), S100A11-knockdown cells did not form tumours, while untreated cells formed tumours in some cases (Fig. 2J, K). Then, the frequency of CSC was calculated, which indicated that S100A11 downregulation significantly reduced its frequency to 1 in every 214,408 cells in BxPC3, whereas 1 in 49,326 control cells had the ability to form tumours. Similar results were shown in SW1990, S100A11 knockdown reduced the CSC frequency to 1 in 164,229 cells, whereas it was 1 in 45,512 control cells (Table 1). In conclusion, these experiments indicated that S100A11 is a critical regulator that promotes malignant properties and that targeting S100A11 is a potentially valuable strategy for treating PDAC.

**Table 1:** ELDA assay in vivo shows that S100A11 significantly increases the stem cell frequency of BxPC3 and SW1990 cells. Stem cell frequency of BxPC3 and SW1990 cells.

BxPC3 Group	Lower	Estimate	Upper	2e + 06 Tested/Response	2e + 05 Tested/Response	2e + 04 Tested/Response
Con	195319	45512	10605	3/3	3/3	3/2
KD	463931	164229	58136	3/3	3/3	3/0
SW1990 Group	Lower	Estimate	Upper	5e + 06 Tested/Response	5e + 05 Tested/Response	5e + 04 Tested/Response
Con	354912	49326	6855	3/3	3/3	3/1
KD	862258	214408	53315	3/3	3/2	3/0

### S100A11 expression was correlated with glutathione metabolism and implicated in PPP

**Fig. 3:**
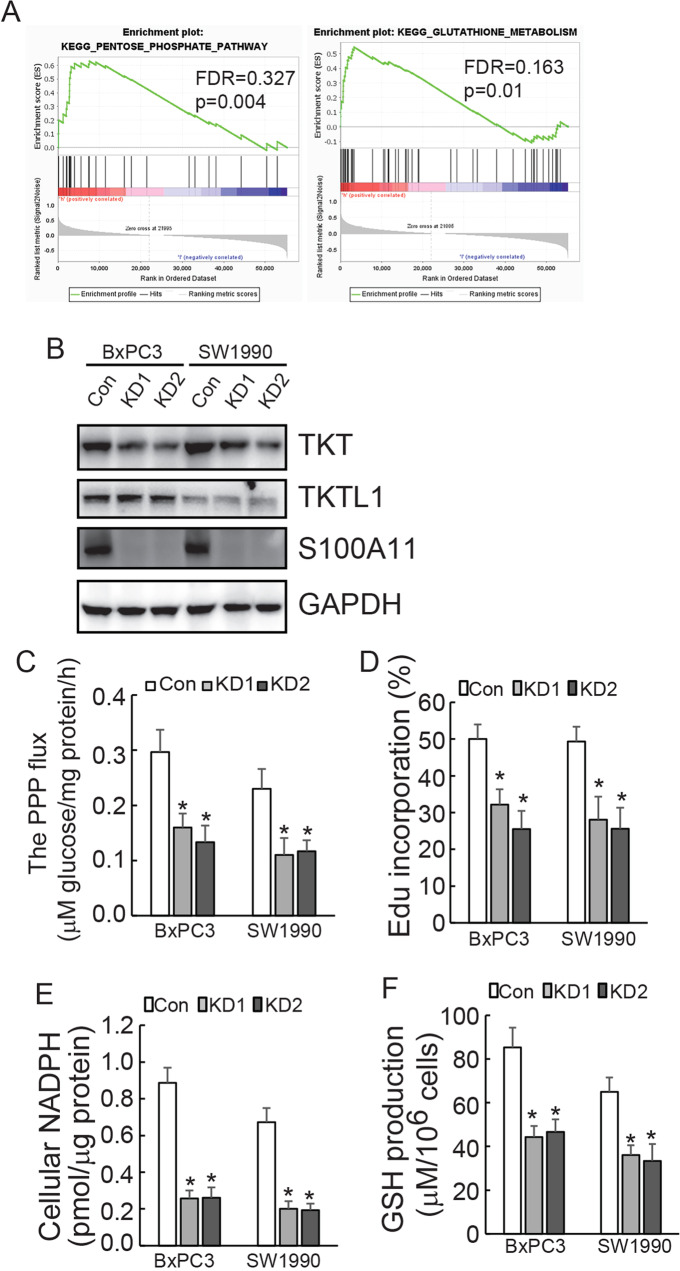
S100A11 knockdown suppresses the PPP in PDAC cells. **A** GSEA indicated that S100A11 was enriched in the PPP and glutathione metabolism in the KEGG dataset. **B** TKT and TKTL1 proteins were analysed using western blotting in control and S100A11-knockdown PDAC cells. **C–F** Relative PPP flux, de novo DNA synthesis, cellular NADPH levels and GSH production were measured and normalised by cellular proteins in PDAC cells after S100A11 knockdown. *p < 0.05. Error bars indicate mean ± SD.

To address S100A11-related signal pathways comprehensively, GSEA showed that S100A11 was significantly enriched in the PPP and glutathione metabolism pathway in pancreatic cancer (Fig. 3A). Furthermore, we utilised BxPC3 cells to perform SILAC-MS, the screening of which indicated that TKT, an enzyme implicated in PPP, ranked as the third most significantly downregulated protein in S100A11-knockdown cells (Supplementary Data 1). The other protein closely related to TKT is transketolase-like protein 1 (TKTL1). TKT is enzymatically active as both a homodimer (TKT-TKT) and a heterodimer (TKT-TKTL1), which induced DNA synthesis and cell cycle progression in a manner of ribose-5-phosphate accumulation [17]. Western blotting demonstrated that S100A11 knockdown has resulted in the decreased expression of TKT without affecting TKTL1 (Fig. 3B). It is suggested that S100A11 may regulate PPP in a TKTL1-TKT heterodimer-independent manner, which highlighting clinically a significant association between S100A11 and TKT in pancreatic cancer.

Based on the biological role of TKT, we assessed whether S100A11 might have any influence on the glucose flux through the PPP. Decreased flux was indeed observed when S100A11 was knocked down in both PDAC cells (Fig. 3C). As the flux through the PPP generates NADPH and ribose-5-phosphate, which are important precursors for DNA biosynthesis, de novo biosynthesis of DNA was measured by Edu incorporation. The findings showed a significant decrease of this biosynthesis in PDAC cells with S100A11 knockdown (Fig. 3D). Consistent with this, clear downregulation of cellular NADPH (Fig. 3E) as well as GSH production (Fig. 3F) was observed in both PDAC cells with S100A11.

### S100A11 promoted malignant properties of PDAC cells by regulating TKT

**Fig. 4:**
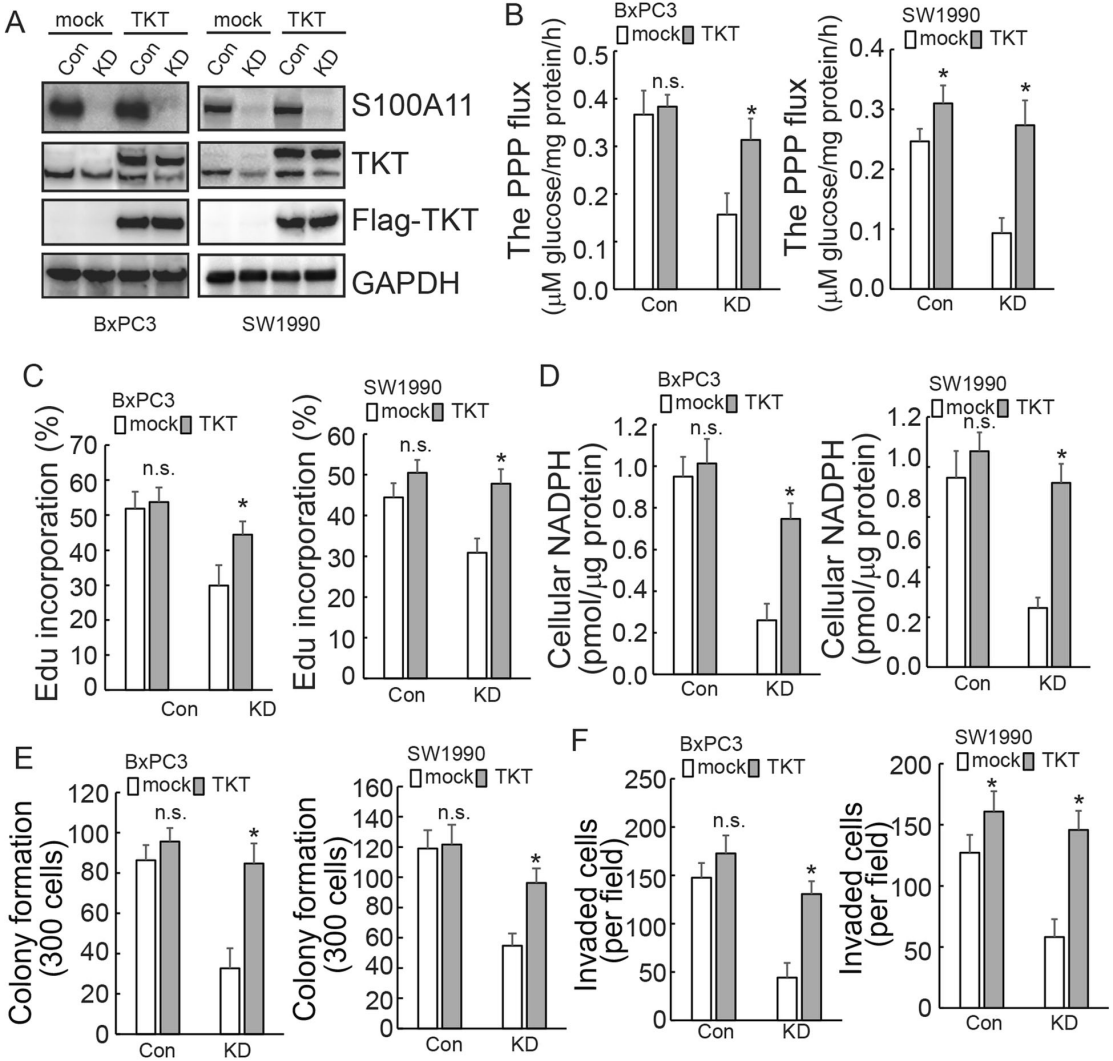
S100A11 promotes the survival and biosynthesis of PDAC cells by regulating TKT expression. **A** Control or S100A11-knockdown PDAC cells were transfected with or without ectopic FLAG-tagged TKT and western blot analysis was performed using the indicated antibodies. **B–D** Control or S100A11-knockdown PDAC cells were transfected with empty or FLAG-tagged TKT plasmid. PPP flux was analysed using NMR. De novo DNA synthesis was analysed using EdU incorporation. Cellular NADPH level was measured in PDAC cells after S100A11 knockdown. **E, F** In the above cells, the effect of TKT on proliferation and migration was confirmed by colony formation and Transwell assays. n.s., not significant. *p < 0.05. Error bars indicate mean ± SD.

The finding that S100A11 decreased the activity of the PPP, of which TKT is a key enzyme, prompted us to investigate whether S100A11 regulates the expression of TKT for the survival and biosynthesis of cancer cells. TKT was ectopically overexpressed in PDAC cells (Fig. 4A). Forced TKT overexpression almost completely recovered PPP flux (Fig. 4B), DNA synthesis (Fig. 4C) and cellular NADPH (Fig. 4D) mediated by S100A11 knockdown. Moreover, the overexpression of TKT contributed to remarkable recoveries in the cells’ colony formation (Fig. 4E) and migration abilities (Fig. 4F) that had decreased due to the decline of S100A11 in PDAC cells.

### S100A11 affected the activity of the *TKT* promoter in PDAC cells

**Fig. 5:**
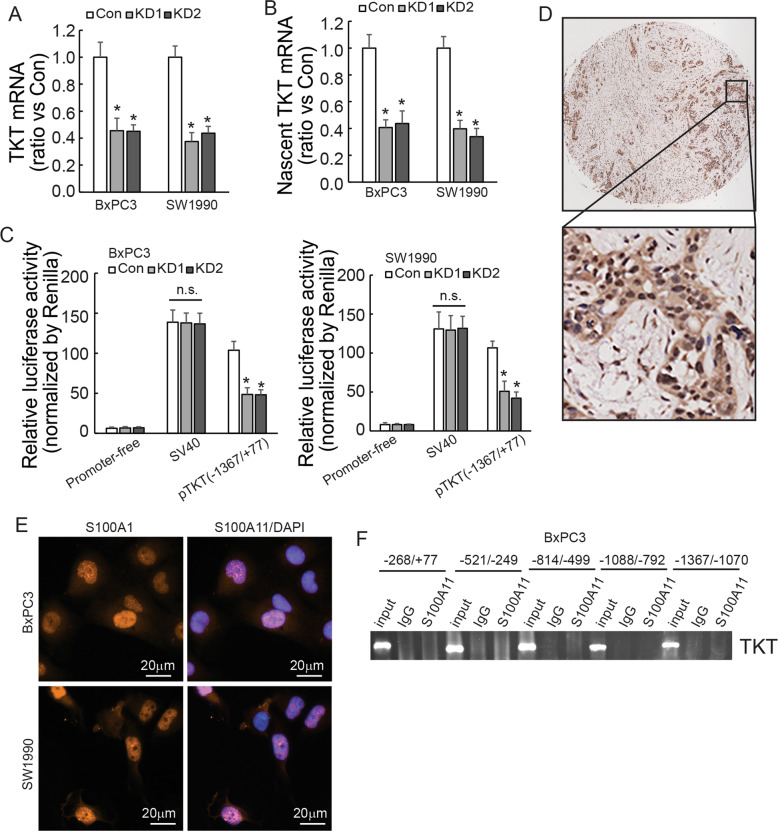
S100A11 affects the activity of the *TKT* promoter. **A** RT-qPCR analysis of TKT mRNA expression in PDAC cells with or without S100A11 knockdown. Samples were normalised to GAPDH mRNA. **B** Transcriptomic analysis of TKT nascent mRNA expression was performed in control and S100A11-knockdown PDAC cells. **C** Relative activity of the *TKT* promoter containing firefly luciferase reporter treated with or without S100A11 knockdown. **D** Representative immunohistochemical staining with S100A11 protein in PDAC tissues. **E** Immunofluorescence showed the subcellular localisation of S100A11 in pancreatic cancer cells. **F** Chromatin prepared from BxPC3 was incubated with anti-S100A11 and IgG antibody. ChIP assay was carried out and the *TKT* promoter in different domains was amplified. *p < 0.05. N.S., not significant. Error bars indicate mean ± SD.

To investigate the potential mechanism underlying the regulation of TKT by S100A11, TKT mRNA expression was first measured by RT-qPCR. Knockdown of S100A11 markedly decreased TKT mRNA expression in both PDAC cells (Fig. 5A). TKT nascent RNA was also decreased in S100A11-knockdown cells (Fig. 5B). Consistent with the above findings, S100A11 knockdown reduced luciferase activity of the *TKT* promoter flanking −1367/+77 (Fig. 5C). These findings indicated that S100A11 regulates TKT at the transcriptional level. Previous reports demonstrated that the S100A family can act as transcription factors to modulate gene expression [18, 19]. S100A11 was found to be strongly expressed in the nucleus in PDAC tissues (Fig. 5D). In parallel with this, immunofluorescence staining showed the same trend in both PDAC cells (Fig. 5E). However, ChIP analysis showed that S100A11 was not recruited to any of the *TKT* promoter regions from −1367/+77 in BxPC3 cells (Fig. 5F), ruling out the possibility that S100A11 functions as a transcriptional regulator to transactivate the *TKT* promoter directly.

### S100A11 binding with SMYD3 promoted the enrichment of H3K4me3 at the *TKT* promoter region

**Fig. 6:**
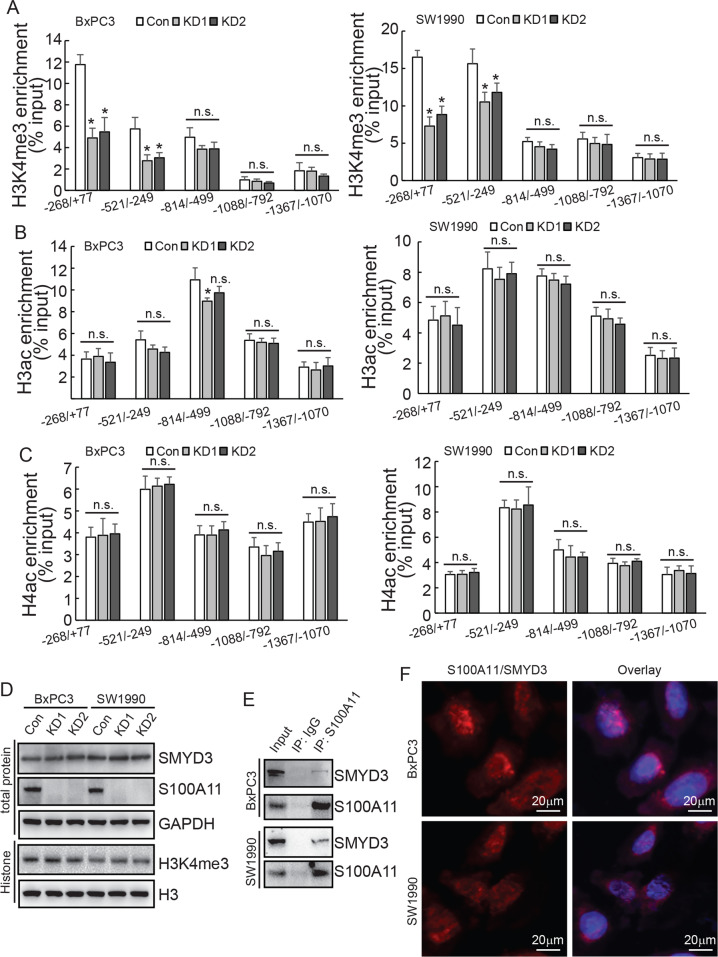
S100A11 promotes H3K4me3 enrichment in the region of the *TKT* promoter by interacting with SMYD3. **A–C** ChIP assays were performed to study the direct affinity of H3K4me3, H3ac and H4ac to the *TKT* promoter region (268/+77, 521/249, 801/503, 1088/792 and 1367/1072) in PDAC cells. **D** Western blotting determined the expression levels of SMYD3 and H3K4me3 proteins after S100A11 knockdown in PDAC cells. **E** Co-IP assay was performed to detect the interaction between S100A11 and SMYD3 in PDAC cells. **F** Direct interaction of endogenous S100A11 with SMYD3 was analysed using DuoLink PLA in BxPC3 or SW1990 cells. *p < 0.05. N.S., not significant. Error bars indicate mean ± SD.

The nuclear localisation of S100A11 indicated that it might indirectly transactivate the *TKT* promoter by interacting with other transcription factors or chromatin-modulating factors. Co-IP followed by MS was performed to screen interactive partners of S100A11 (Supplementary data 2). Histone methyltransferases (HMTs) SMYD3, DOT1L, EHMT1, and SETD7, as well as acetyltransferases KAT2A and KAT5, were among the potential S100A11 interacting partners. Numerous experimental studies have suggested that the regulation of gene expression by histone methyltransferases and acetyltransferases is closely associated with cancer development [20, 21]. In this context, ChIP assay was performed to analyse the epigenetic regulatory mechanism of S100A11-knockdown cells. The results showed that there was reduced enrichment of H3K4me3 at the *TKT* promoter region in the −268/+77 and −521/−249 regions, but this was unaltered in other regions (Fig. 6A). In addition, ChIP assays showed no effect on H3ac and H4ac protein enrichment in the above five promoter sites (Fig. 6B, C). Moreover, the enrichment of mono-, di-, or tri-methylation of H3K79 to the *TKT* promoters was unaltered by S100A11 knockdown (data not shown). All of these results indicated that S100A11 might regulate the recruitment of H3K4me3 to the −268/+77 and −521/−249 regions of the *TKT* promoter, thereby affecting TKT transcription. SMYD3 is a histone lysine methyltransferase that plays an important role in transcriptional activation. It was described as a histone H3K4-specific di- and tri-methyltransferase exerting its oncogenic effects through the transcriptional activation of downstream target genes [22, 23]. Western blotting demonstrated that the expression of intracellular SMYD3 protein and total H3K4me3 levels remained unchanged after S100A11 downregulation (Fig. 6D). However, subsequent Co-IP assay revealed that S100A11 could interact with SMYD3 (Fig. 6E). In addition, PLA assay further demonstrated direct interaction of endogenous S100A11 and SMYD3 (Fig. 6F). These findings suggested that S100A11 may alter H3K4 methylation at the *TKT* promoter region by interacting with SMYD3.

### SMYD3 specifically bound to the *TKT* promoter at the −268/+77 region

**Fig. 7:**
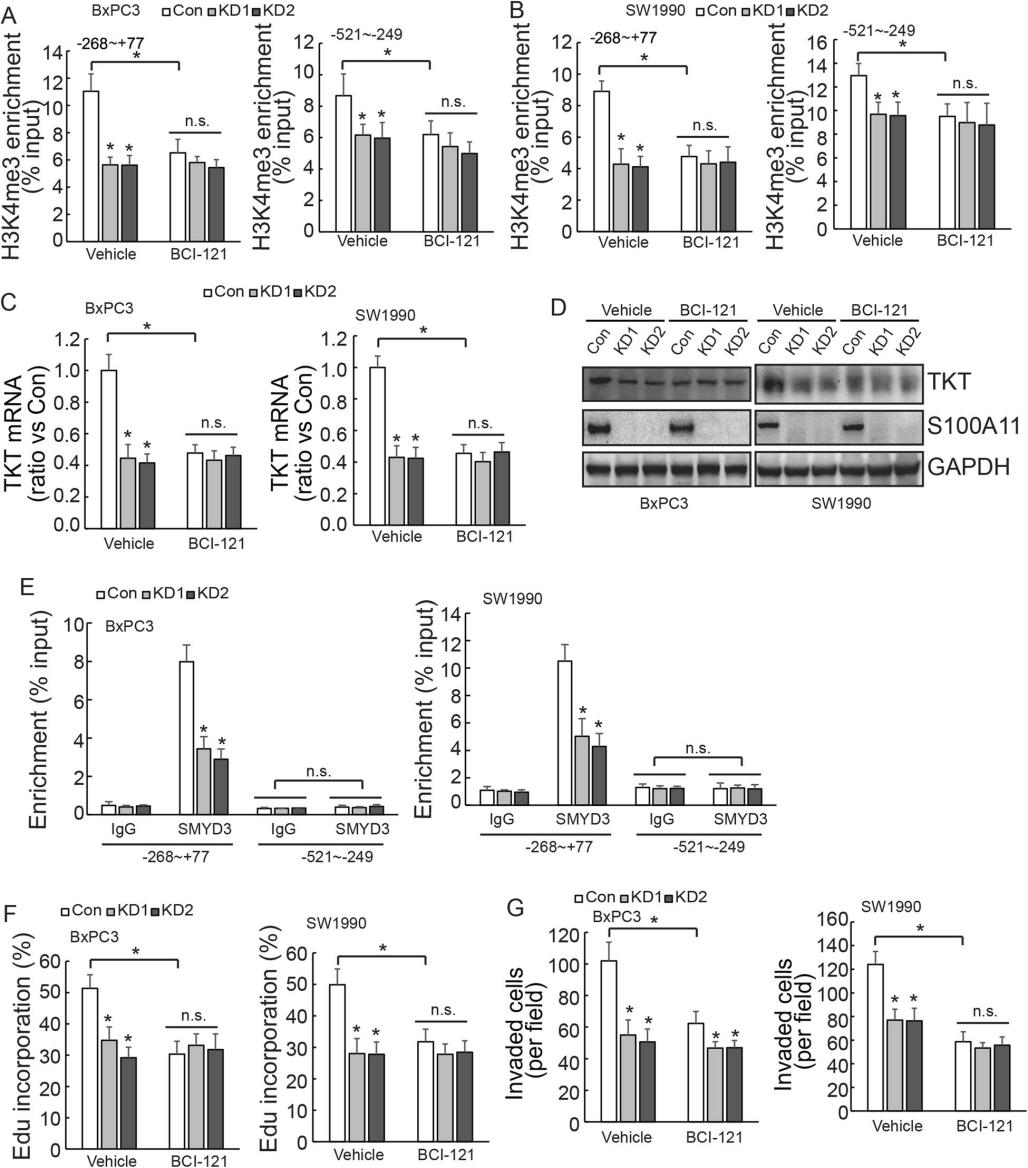
SMYD3 specifically binds to the *TKT* promoter to promote tumour progression. **A**, **B** ChIP assay was performed to compare the H3K4me3 enrichment of cells with or without BCI-121 treatment in S100A11-knockdown or control PDAC cells. **C** TKT mRNA expression with or without BCI-121 was analysed using RT-qPCR in S100A11-knockdown or control PDAC cells. **D** Western blotting determined the expression level of TKT protein with or without BCI-121 in S100A11-knockdown or control PDAC cells. **E** ChIP assay was performed to compare the SMYD3 enrichment after S100A11 knockdown in PDAC cells. **F** Control or S100A11-knockdown cells were treated with vehicle or BCI-121, and DNA synthesis was analysed using EdU incorporation. **G** Transwell assay was performed to compare the invasiveness of cells with or without BCI-121 in S100A11-knockdown or control PDAC cells. *p < 0.05. N.S., not significant. Error bars indicate mean ± SD.

BCI-121 is a competitive inhibitor of SMYD3. ChIP assay further confirmed that BCI-121 significantly reduced the enrichment of H3K4me3 in the −268/+77 and −521/−249 regions of the *TKT* promoter in control cells, while having less of an effect in both PDAC cells with S100A11 knockdown (Fig. 7A, B). Similar results were observed for TKT mRNA and protein expression (Fig. 7C, D). ChIP demonstrated that SMYD3 was only enriched in the −268/+77 region of the *TKT* promoter, and the enrichment rate was markedly decreased after S100A11 knockdown. SMYD3 was not obviously enriched in the −521/−249 region of the *TKT* promoter (Fig. 7E). BCI-121 treatment significantly reduced DNA synthesis and invasiveness in control cells, while having little effect in cells with S100A11 knockdown (Fig. 7F, G). All of these findings indicated that S100A11 might promote H3K4me3 protein enrichment in the −268/+77 region of the *TKT* promoter and activate TKT transcription, thereby promoting the proliferation and invasion of PDAC cells by interacting with SMYD3.

## Discussion

S100A11 is a member of the S100 family, which exerts its biological functions as a calcium sensor or binding protein [24]. Recently, studies have found that, the expression of S100A11 is positively correlated with the degree of cancer progression and an unfavourable clinical prognosis [13, 25, 26]. Silencing of S100A11 in intrahepatic cholangiocarcinoma can reduce the level of SMAD2/3 phosphorylation which is induced by TGF-β1, and then inhibit cell migration, invasion and epithelial-mesenchymal transition (EMT) [27]. Moreover, S100A11/ANXA2 complex is overexpressed in stressed cells to maintain the integrity of the plasma membrane, which can be damaged by cancer cell migration in hepatocellular carcinoma (HCC) [28]. S100A11 is widely overexpressed in pancreatic cancer tissues [14]. Our study showed that S100A11 was mostly expressed in the nucleus and cytoplasm, but predominantly cytoplasm in previous report [29]. We supposed that the expression difference might be related to the physiological environment under diverse clinicopathological conditions. In human keratinocytes, elevation of extracellular Ca2+ concentration caused S100A11 interacts with Annexin I, S100A11 can be redistributed from cytoplasm to cell periphery [30]. S100A11 may performs its biological functions through dynaminc binding to specific proteins and changing their activity or cellular location under diverse physiological circumstances. The mechanism would be gradually revealed with the exploration of S100A11. In the current study, we demonstrated that S100A11 expression positively correlated with unfavourable prognosis in PDAC patients. Downregulation of S100A11 significantly reduced malignant behaviour by decreasing the activity of the PPP. The findings showed a metabolic mechanism for the role of S100A11 in promoting PDAC tumorigenesis and development.

The PPP, as a branch of glycolysis for maintaining biosynthesis and controlling redox homoeostasis, is a fundamental component of cellular metabolism [5, 6]. It has been reported that enhanced activity of the PPP provides materials for anabolism like pentose phosphates, to use their high rate of biosynthesis, including DNA synthesis, which is considered to be critical for tumour progression and drug resistance [5, 31–34]. In our study, GSEA revealed that S100A11 associated with PPP and glutathione metabolism pathway. In addition, quantitative proteomics identified that TKT was the gene whose expression was most altered upon S100A11 downregulation. As a key enzyme in the non-oxidative PPP, the expression and activity of TKT endow the PPP with the ability to maintain a dynamic reaction to switch the metabolic pattern in adaptation to the microenvironment [5, 35].

Previous reports have indicated that an inhibitor of TKT, oxythiamine, decreased cell proliferation through inhibition of the pentose cycle and induced a G1 phase arrest in tumour cells [34, 36, 37]. Therefore, targeting TKT metabolic pathways introduce a new clue for the development of future cancer therapies. In our study, downregulation of S100A11 suppressed the PPP by decreasing TKT expression and affected the malignancy behaviour of PDAC. Previous reports showed that TKT inhibition decreased PPP flux and increased sensitivity to gemcitabine and imatinib in tumour cells [32, 33]. It demonstrated that increased PPP generates biosynthetic intermediates to accelerate tumours repair, which resulting in resistance. Whether S100A11 affects the sensitivity of chemotherapy by regulating TKT could be further confirmed and discussed in future studies.

Although accumulating evidence has confirmed that TKT promotes the proliferation and migration of various cancer cells [38–40], the specific biological mechanisms underlying the regulation of TKT mediated by S100A11 remain incompletely understood. Our study demonstrated that S100A11 knockdown primarily affected novel synthesis of mRNA. Furthermore, the expression of S100A11 could affect *TKT* promoter activity in the region of −1367/+38. Generally speaking, the activation or inhibition of gene transcription is usually accomplished by regulation via transcription factors and epigenetic modifications [41, 42]. It has been reported that the S100 protein family possesses a wide range of biological functions [43, 44]. Specifically, S100A2/A4/A6 can interact with the transcription factor p53 to activate or interfere with its transcriptional activity [45–48]. Moreover, S100A4 also activates the transcription factor NF-κB and promotes the transcription of multiple genes [49, 50]. Our study surprisingly revealed that S100A11 is highly expressed in the nucleus, but ChIP assay strongly suggesting that S100A11 protein could not bind to the *TKT* promoter directly in the region of −1367/+38. Our findings thus indicated that S100A11 may regulate the transcription of TKT through other covert mechanisms.

In view of the above results, we conducted an elaborate analysis of another potential key regulatory mechanism that may affect TKT expression. Epigenetic modifications such as DNA methylation, histone modification and chromatin remodelling have been shown to have a considerable influence on gene expression. Histone modifications have been intensely studied for their roles in the pathogenesis of various diseases as they cause a shift in transcriptional regulation [51, 52]. Numerous experimental studies have revealed that the regulation of gene expression by histone methyltransferases and acetylases plays an important role in cancer development and metastasis [47, 53, 54]. H3K4me3, H3ac and H4ac as histone modifications are characteristic marks associated with active transcription [55]. In the current study, we detected that significant decrease of H3K4me3 enrichment in some regions of the *TKT* promoter after the downregulation of S100A11, while H3ac and H4ac were not significantly affected. This suggests that S100A11 might affect TKT gene expression through methylation rather than acetylation of histones.

The methyltransferase SET/MYND family proteins (SMYD1–5) are specifically responsible for H3K4 methylation [56–58]. Among these, SMYD3 can methylate non-histone targets, thereby enhancing the associated kinase activity [59]. These findings revealed a key role of SMYD3 in determining carcinogenesis and development of cancer cells. Our study observed abundant protein binding between SMYD3 and S100A11. BCI-121 suppressed the enrichment of H3K4me3 at the TKT promoter site in control cells, and TKT mRNA and protein expression, while it did not change in cells with S100A11 knockdown. Furthermore, BCI-121 also clearly suppressed the proliferation and invasion of control cells. Notably, SMYD3 only bound to the −268/+77 region of the *TKT* promoter, but not to the −521/−249 region, although H3K4me3 levels at both sites were decreased by S100A11 knockdown. HSP90 is critical for the nuclear localisation of SMYD3 [60]. Mutated SMYD3 was shown to lead to decreased binding of HSP90 to SMYD3, which in turn led to decreased methylation of H3K4 [61, 62]. This suggests that HSP90 binding to SMYD3 not only assists in localisation, but is also necessary for the methyltransferase activity of SMYD3. In this way, the differential expression of HSP90 might affect the binding of SMYD3 to the promoter region of target genes, although this requires further verification. Alternatively, besides SMYD3, there may be other methyltransferases that can bind with S100A11 to promote the enrichment of H3K4me3 in the *TKT* promoter region. All of these issues require further investigation in future study.

In summary, the current study demonstrated that S100A11 expression increased in PDAC and was significantly associated with worse prognosis and disease progression. Mechanistically, we showed that downregulation of S100A11 suppressed the PPP by inhibiting TKT transcription and decreased H3K4me3 loading to the *TKT* promoter by impairing the interaction with SMYD3 protein. Enhanced TKT biosynthesis activated the PPP in S100A11-knockdown cells, subsequently promoting the proliferation and invasion of PDAC cells. Therefore, given the relevance of S100A11 for the malignant behaviour of PDAC cells, as well as its involvement in regulating PPP, S100A11 may be a valuable target for treating PDAC.

## Materials and methods

Commonly biological methods are illustrated in supplementary methods.

### Immunohistochemistry (IHC)

The TMA sections (Shanghai Outdo Biotech Co., Ltd.) were incubated with anti-S100A11 antibody. The immunostaining score was calculated by multiplying the proportion of positively stained cells (0%–100%) by the staining intensity (0: no staining; 1, weak staining; 2, moderate staining; 3, strong staining), as previously reported [63].

### Cell culture

Pancreatic cancer cells BxPC3 and SW1990 were purchased from the Cell Resource Center of Shanghai Institute for Biological Sciences (Chinese Academy of Sciences, Shanghai, China). Cells were cultured in DMEM (Sigma-Aldrich) supplemented with 10% FBS (Sigma-Aldrich). PDAC cells were infected with S100A11 CRISPR/Cas9 knockdown (KD) lentivirus involving a dual gRNA approach, or transfected with ectopic TKT expression vector. Detailed description illustrated in supplementary methods.

### Sphere formation assay

Single-cell suspensions of PDAC cells were grown as spheres in serum-free DMEM supplemented with B27 (Life Technologies), 20 ng/mL EGF, and 20 ng/mL FGF. Viable cells were diluted to the indicated cell number in 100 μL of medium/well in a 96-well plate after growth at 37 °C and 5% CO_2_. The cells with a diameter ≥ 50 μm were counted.

### Animal experiments

Five-week-old BALB/c-nu/nu female mice were purchased from Shanghai Laboratory Animal Center (Chinese Academy of Science). All animal procedures were approved by the Institutional Animal Care Committee of China Medical University and complied with its guidelines. Mice were randomly assigned to subcutaneous injection with S100A11 knockdown or control cells (*n* = 3). Six weeks after injection, mice were scored for the presence of palpable tumours. The dose of cells injected into the mice without tumours was plotted and the slope of the graph was used to estimate the proportion of stem cells using extreme limiting dilution analysis (ELDA) software.

### SILAC-MS

Control and S100A11-knockdown BxPC3 cells were labelled with Light and Heavy Isotopic Labels, respectively. Cells were grown in the medium supplemented with 200 µg/ml l-proline to achieve complete incorporation (>99%) of the respective labels. Cells were lysed and 0.1% benzonase was added to digest nucleic acids. Cell lysates were cleared and 2D Quant Kit was used to measure protein concentrations. Samples were analysed by nanoLC-MS/MS with LTQ-Orbitrap. Peptide samples were separated on a C18 column after being desalted on a trap column. The mobile phase A was 0.1% formic acid in HPLC-grade water, and 0.1% formic acid in ACN for mobile phase B. The peptide mixture was separated at 2 μl/min with a linear gradient of 4%-50% B for 110 min, following by 50%-100% B from 110 min to 115 min, then maintained at 100% B for 5 min. Data-dependent MS/MS mode was used, in which each scan cycle consisted of one full MS scan in profile mode followed by seven MS/MS scans in centroid mode.

### Co-immunoprecipitation and western blotting

Total cellular proteins (3 mg) from PDAC cells were extracted using lysis buffer containing 20 mM Tris-HCl, 150 mM NaCl, 2 mM EDTA, 1% Triton X-100, and protease inhibitor cocktail (Sigma-Aldrich). Protein-A/G beads (Biomake) were pre-incubated with lysates and centrifuged, 2 μg of S100A11 antibody or IgG was added to the lysates, followed by storage overnight at 4 °C. Next, 25 μl of Protein-A/G beads were added and kept on a rotator for 2 h at 4 °C. Then, immunoprecipitates were analysed by western blotting. Extracted proteins were quantified using the BCA protein assay kit (Thermo Scientific). Twenty micrograms of total protein was separated using 10%–12% SDS-PAGE and transferred to PVDF membrane (Millipore). The membranes were incubated in protein blocking solution and probed with primary antibody.

### Measurement of PPP flux

The flux through the oxidative branch of the PPP was assessed with carbon-13 nuclear magnetic resonance spectrometry. Control and S100A11-knockdown PDAC cells transfected with or without exogenous TKT expression vector were cultured to approximately 60–70% confluence. After washed with no glucose medium, the cells were incubated with 10 mM [2-^13^C] glucose medium for 9 h. The rates of total glucose consumption and lactate formation were determined with a bench top analyzer (Nova Biomedical, Waltham, MA). The final medium from each culture was analysed in a 20-mm NMR tube with a 9.4 Tesla spectrometer (Varian, Palo Alto, CA) at 100.66 MHz. A 90°excitation pulse was applied every 6 s (fully relaxed), with broadband decoupling used only during ^13^C data acquisition (no NOE enhancement). Spectra were acquired with 32768 points, a spectral width of 25,000 Hz and 3000 excitations. Free induction decays were apodized with exponential multiplication (1 Hz line broadening). The ratio of ^13^C in carbons 3 (reflecting oxidative PPP flux) of lactate and the rate of glucose consumption were used to estimate the oxidative PPP flux.

### Cellular NADPH measurement

Cellular NADPH concentrations were measured using NADPH assay kit (Abcam). Briefly, PDAC cells were lysed with 400 μl of NADP^+^ extraction buffer and heated at 60 °C for 30 min. Twenty microliters of assay buffer and 200 μL of NADPH extraction buffer were added to neutralise the extracts. The extracts were used to determine the NADPH concentration. The absorbance of the reaction mixture at 565 nm was measured by a plate reader at 0 and 30 min.

### Quantification of GSH

In an attempt to analyse extracellular GSH levels, PDAC cells were seeded in 24-well plates. After incubation overnight, cells were primed with LPS (100 ng/mL) for 5 h before being washed with l-methionine- and l-cystine-free DMEM (Sigma-Aldrich) supplemented with 2 mM L-glutamine, 10% foetal bovine serum and 1% penicillin-streptomycin. Then, ATP-containing complete l-methionine- and l-cystine-free DMEM was added to the cells to prevent reactions between culture medium components and GSH or its metabolites. After incubation, culture supernatants and cells were harvested and analysed by LC–MS/MS.

### Labelling and capture of nascent RNA

Nascent synthesised RNA was labelled and captured using the Click-iT Nascent RNA Capture kit (Thermo Fisher). PDAC cells were cultured with 5-ethymyl uridine (EU) for 4 h at a final concentration of 200 μM and incubated for 30 min during DMSO or CHX treatment. Cells were washed, harvested by trypsinisation and counted. Total RNA was isolated, subjected to nascent RNA capture and analysed by real-time RT-PCR.

### In situ proximity ligation assay (PLA)

PLA were conducted using the Duolink PLA kit (Sigma-Aldrich). PLA probe antibodies were directed against the primary antibodies for S100A11 and SMYD3. Briefly, PDAC cells were seeded onto a slide, washed with PBS buffer and fixed with 4% paraformaldehyde for 20 minutes at room temperature. The cells were permeabilized with 0.1% Triton X‐100 for 20 minutes at room temperature. The primary antibodies used in this study were anti‐S100A11, anti‐SMYD3. The PLA probes were anti‐mouse (minus) and anti‐rabbit (plus). Duolink in situ Detection Reagent Red was used for detection. Imaging was performed using a confocal fluorescence microscope. Images were analysed with Image‐Pro Plus software. At least 50 cells were analysed in each experiment.

### Chromatin immunoprecipitation (ChIP)

ChIP assay was performed using a kit from Upstate Biotechnology Inc. The cells were fixed with 1% formaldehyde to crosslink chromatin and lysates were sonicated on ice. PDAC cells were respectively transfected with the S100A11, H3K4me3, H3ac, H4ac, SMYD3 antibodies and a negative control IgG antibody. Immune complexes were collected with salmon sperm DNA-saturated protein A‐agarose beads. IP complexes were eluted with 0.1 mol/L NaHCO_3_ and 1% SDS. The protein-DNA crosslinks were reversed by incubating at 65 °C for 5 h. DNA crosslinked with S100A11 was amplified by PCR with TKT-specific primers. The PCR products were analysed by agarose gel electrophoresis and visualised with ethidium bromide under ultraviolet light. The DNA crosslinked with H3K4me3, H3ac, H4ac, SMYD3 was amplified by quantitative real-time PCR. Results were shown as the ratio of purified DNA products to input product.

### Statistics

Statistical analyses were performed using ANOVA and post hoc Dunnett’s test. Statistical significance was defined as *p* < 0.05. All data were obtained from three independent experiments and are presented as the mean ± standard deviation (SD).

## Supplementary information

Supplementary data 1

Supplementary data 2

Supplementary method

Reproducibility checklisś

Original Western blot figures
