# Supplementary material for: S100A11 activates the pentose phosphate pathway to induce malignant biological behaviour of pancreatic ductal adenocarcinoma
Source: Cell Death Dis. 2022 Jun 25;13(6):568. doi: 10.1038/s41419-022-05004-3 (PMC9233679; doi:10.1038/s41419-022-05004-3)
Supplement: Supplementary file 3 — Supplementary method [file 41419_2022_5004_MOESM3_ESM.docx]

**Supplementary methods**

**Supplementary table 1:Antibodies information**

| Proteins | Manufacturer | Cat. |
| --- | --- | --- |
| S100A11 | Proteintech | 10237-1-AP |
| SMYD3 | Proteintech | 66330-1-Ig |
| TKT | Proteintech | 11039-1-AP |
| H3K4Me3 | ThermoFisher | 711958 |
| Flag | CST | 86861 |
| H3 histone | CST | 9715 |
| GAPDH | Abcam | ab9485 |
| H3ac | ThermoFisher | PA5-114693 |
| H4ac | ThermoFisher | PA5-40083 |
| IgG | CST | 2729 |

**Supplementary table 2: S100A11 CRISPR/Cas9 knockdown and negative control lentivirus sequences and primers**

| Gene |  | Sequence |
| --- | --- | --- |
| S100A11 | Negative control | ACTGAGCGGTGCATCGAGTC |
|  | gRNA#1 | ACTGAGCGGTGCATCGAGTC |
|  | gRNA#2 | GCTGTCTTCCAGAAGTATGC |
|  | gRNA#3 | AGACAGAGTTCCTAAGCTTC |
| S100A11 primer | forward | TCCAAGACAGAGTTCCTAAG |
|  | reverse | AGGCCACCAATCAGATTAAG |

**Supplementary table 3: Primers used to amplify the TKT promoter**

| Region |  | Sequence |
| --- | --- | --- |
| -268/+77 | forward | 5’-CAAGTCCACGGAGGACTGTG-3’ |
|  | reverse | 5’-ACACACACAGAGATAGCGGCTG-3’ |
| -521/-249 | forward | 5’-TCCCAGCTACTAGGGAGGCTG-3’ |
|  | reverse | 5’-CACAGTCCTCCGTGGACTTG-3’ |
| -814/-499 | forward | 5’-GAAGGATCACTTGAGCCCAG-3’ |
|  | reverse | 5’-TCAGCCTCCCTAGTAGCTGG-3’ |
| -1088/-792 | forward | 5’-ATCACCTGGCATTGTCATGG-3’ |
|  | reverse | 5’-CTCCTGGGCTCAAGTGATCC-3’ |
| -1367/-1070 | forward | 5’-TCTTCCTCTGTAAAATAGGC-3’ |
|  | reverse | 5’-CATGACAATGCCAGGTGATG-3’ |

**Stable cell line establishment**

Although S100A11 expression can be detected in both PDAC cells, to better persuasive, we used the S100A11 control sequence as a negative control considering the transfection toxicity of CRISPR/Cas9 system. 1 × 10^5^ cells/well were seeded into six-well plates and cultured for 24 h. The dual gRNA construct carrying Cas9 and the donor vector were infected into PDAC cells in accordance with the manufacturer’s instructions (Genechem). Viral solutions according to the multiplicity of infection (MOI) were added into cell culture medium. After incubation for 8 h, the medium were changed by normal medium. To sort positive clones, GFP and PU were integrated into the genomic DNA by homologous recombination. The efficiency of gRNA was determined by real-time RT-PCR and western blot.

TRIzol reagent was used to extract total RNA from Hela cells. Then, the RNA was reverse-transcribed to cDNA with a reverse transcription kit for reverse-transcription polymerase chain reaction (RT-PCR). TKT cDNA was amplified and cloned into the pCMV-3×FLAG 7.1 (Sigma–Aldrich), and pCMV-3×FLAG-BAP was used as a negative control. The TKT plasmid was extracted with a QIAprep spin Miniprep Kit (QIAGEN). Ectopic TKT expression vector and its corresponding control was transfected employing the Lipofectamine 2000 reagent (Thermo Fisher Scientific) following the manufacturer’s instructions. After 48 h transfection, the expression of target protein and gene were evaluated by Western blot.

**Survival analysis**

Briefly, users input the gene of S100A11 and select pancreatic adenocarcinoma (PAAD) for the survival analyses in the GEPIA website (http://gepia.cancer-pku.cn/index.html), GEPIA uses log-rank test for the hypothesis evaluation. The cox proportional hazard ratio and the 95% confidence interval information can also be included in the survival plot.

**Quantitative real-time PCR analysis**

Total cellular RNA was isolated with Trizol. RNA reverse transcriptions of messenger RNA (mRNA) was performed using SuperScript III Reverse Transcriptase (Thermo Fisher). The cDNA was amplified with SYBR Premix Ex Taq II Kit (Takara) and measured using the ABI 7500 RealTime PCR Detection System (Applied Biosystems). The following cycle parameters were used: 95°C for 30 s; 45 cycles at 95°C for 5 s and 58°C for 34 s. The data analysis was evaluated via the sample threshold cycle (Ct) value, and the relative change in each sample was normalized to internal control gene. The fold change of target RNA expression was calculated using the 2^-ΔΔCt^ method.

**Cell proliferation assay**

Cells were seeded into six-well plates at a density of 100,000 per well in 2 ml of medium supplemented with 10% FBS. The medium was changed every day. The dead cells were stained with trypan blue at the indicated time points and their number was determined by counting using an automated cell count kit.

**Colony formation assay**

Cells were seeded at 400 cells/mL in six-well plates for 10 days and the medium was replaced with fresh medium every 3 days. Cells were stained with crystal violet. Colonies containing ≥ 50 cells were counted.

**Transwell invasion assay**

The invasiveness of cells was analysed using the Boyden Chamber (BD Biosciences) assay. Briefly, the Transwell membranes were coated with Matrigel and DMEM containing 10% foetal bovine serum was added to the lower chamber. Cell solution containing serum-free medium was added on top of the Matrigel coating membrane. After 24 h of incubation, the filter was gently removed from the chamber and migrating cells were stained with crystal violet.

**Gene Set Enrichment Analysis (GSEA)**

Briefly, login the GDC Data Portal (https://portal.gdc.cancer.gov/) to download pan-cancer tumour gene expression data sets. Next download the S100A11 functional gene set (http://www.gsea-msigdb.org/gsea/downloads.jsp). The biological functions of the S100A11-driven genes were clarified using GSEA (http://software.broadinstitute.org/gsea/index.jsp). The degree of enrichment and statistical significance were quantified using normalised enrichment score (NES), nominal *P*-value, and FDR. Kyoto Encyclopedia of Genes and Genomes (KEGG) pathway analysis was conducted using GSEA software v3.0 (www.broadinstitute.org/gsea).

**EdU incorporation assay**

De novo DNA synthesis was determined using Click-iT Edu Assay Kit (Invitrogen). Briefly, PDAC cells were incubated with 10 μM EdU for 4 h. After fixation, incorporated Edu was labelled with Alexa Fluor 555 azide in the provided reaction buffer for 30 min, and the nuclei were counterstained with DAPI.

**Immunofluorescence assay**

Cells were fixed with 4% paraformaldehyde/phosphate-buffered saline, permeabilised using 0.5% Saponin and 0.5% Triton X-100, and blocked with 3% bovine serum albumin (Roche), prior to indirect immunofluorescence labelling. Unconjugated primary antibodies were used at 1:400 dilution. Confocal imaging of labelled cells was performed using a TCS SP5 X supercontinuum microscope (Leica Microsystems).

**Dual-luciferase reporter assay**

The *TKT* promoter flanking the −1367/+77 region was inserted into the promoter-free pGL4 luciferase vector (Promega). SV40 (positive control) and *TKT*-luc were co-transfected with an internal control, *Renilla* luciferase vector (Promega), into PDAC cells. Luciferase activity was detected overnight after transfection by the Dual-Luciferase Reporter Assay System (Promega), in accordance with the manufacturer’s instructions. Firefly luciferase (FFL) activity was normalised by *Renilla* (RL) activity, yielding the relative activity (RLU).
